# Outcome of Neonatal Hydronephrosis, a New Cut-Off to Identify Patients with Spontaneous Resolution

**DOI:** 10.3390/children11121437

**Published:** 2024-11-26

**Authors:** Antonio Gatto, Serena Ferretti, Arianna Turriziani Colonna, Lavinia Capossela, Antonio Chiaretti, Marcello Covino, Claudia Rendeli

**Affiliations:** 1 Department of Pediatrics, Fondazione Policlinico Universitario “A. Gemelli” IRCCS, 00168 Rome, Italy; serena.ferretti1@guest.policlinicogemelli.it (S.F.); ariannaturrizianic@gmail.com (A.T.C.); laviniacapossela@gmail.com (L.C.); antonio.chiaretti@policlinicogemelli.it (A.C.); claudia.rendeli@policlinicogemelli.it (C.R.); 2 Department of Pediatrics, Università Cattolica del Sacro Cuore, 00168 Rome, Italy; 3 Department of Emergency, Fondazione Policlinico Universitario “A. Gemelli” IRCCS, Università Cattolica Sacro Cuore, 00168 Rome, Italy; marcello.covino@policlinicogemelli.it

**Keywords:** anteroposterior renal pelvis diameter, children, congenital anomalies of the kidney and urinary tract, ultrasound

## Abstract

Background/Objectives: The anteroposterior renal pelvis diameter (APRPD) is used to assess the grade of urinary tract dilatation (UTD). There is no univocal method stratifying the risk of complications related to postnatal UTD. This study aims to identify APRPD cut-offs at birth to determine outcome stratification and second-level exams. Methods: The records of a cohort of newborns with unilateral or bilateral UTD confirmed or detected by ultrasound after birth between 2010 and 2020 were analyzed. These children underwent further examinations at 3, 6, 12, and 24 months of age. Results: We managed 500 children with postnatal UTD, with a median APRPD at 0–2 months of age of 7.7 mm [IQR 6.0–10.0]. As for UTD resolution, 279 (55.8%) patients had a complete resolution at the age of 6 months; an additional 55, for a total of 344 (68.8%), at 9–12 months; and 19, for a total of 353 (70.6%), at 24 months. An APRPD value ≤ 8.5 mm showed a sensitivity of 80.4% (95% CI [76.0–84.4]) and a specificity of 100.0% (95% CI [76.8–100.0]) in identifying candidates for spontaneous resolution within 24 months of life. An APRPD value ≤ 8.5 mm was also an independent prognostic factor of resolution at the age of 24 months (*p* = 0.000). Conclusions: Isolated hydronephrosis is the most frequent urinary tract abnormality detected in pregnancy. A well-structured prenatal and postnatal management plan is indeed necessary. According to our analyses, 8.5 mm can be used as a cut-off to reassure parents and clinicians of the benignity of the postnatal dilatation.

## 1. Introduction

Congenital anomalies of the kidney and urinary tract (CAKUTs) are the most common abnormalities in fetal development. Prenatal detection of urinary tract dilatation (UTD) occurs in 1–2% of all pregnancies subjected to ultrasound scans [1].

The antenatal finding of UTD in most cases is physiologic or transient, without any clinical significance; many of these UTDs are no longer detectable by ultrasound after birth (the best time for postnatal ultrasound is at least 48 h after delivery, in order to avoid underestimation) [2]. When UTDs persist after birth, some resolve autonomously during the first month of life, while others are associated with a specific uropathy such as vesicoureteral reflux, ureteropelvic junction obstruction, posterior urethral valves, or ureterovesical junction obstruction.

Although various scientific societies have proposed a unified grading system [3], several methods for measuring the grade of UTDs are currently used. One of the most used is the quantitative measurement of the anteroposterior renal pelvis diameter (APRPD).

In the literature, much effort has been made to codify the prenatal ultrasound assessment of UTDs, whilst few studies exist on postnatal assessment to define which children with urinary tract dilation require a periodic evaluation and its timing [4].

Although we are aware that moderate-to-severe UTDs are more often linked to worse outcomes and mild forms largely spontaneously resolve, a univocal method for measuring, classifying, and stratifying the risk of complications related to postnatal UTD, as well as a detailed follow-up plan shared by all scientific societies, has not been published yet.

The primary purpose of this retrospective study is to provide useful data about the prognosis of neonatal hydronephrosis from a large cohort of children, in order to identify the cut-offs in the measured values of APRPD at birth to determine the outcome risk stratification and second-level exams.

## 2. Materials and Methods

This is a retrospective study carried out in a cohort of newborns with CAKUTs evaluated in the Spina Bifida and Congenital Uropathies Outpatients Center—Fondazione Policlinico A. Gemelli IRCCS, Rome, Italy, between January 2010 and December 2020.

We managed neonates with unilateral or bilateral UTD detected during pregnancy and confirmed after birth or diagnosed at a US scan performed for other reasons after birth, classified according to the parameters described by Coelho et al. [5], based on the APRPD. The APRPD was classified as mild (5–9.9 mm), moderate (10–14.9 mm), or severe (≥15 mm); having an APRPD value < 5 mm was regarded as a complete resolution of the UTD.

The study population underwent at least two ultrasound (US) examinations of the urinary tract within the first month after birth. Between the two US scans performed within the first month of life, we considered the maximum APRPD measured. Children without evidence of hydronephrosis on US examination were excluded.

Neonates with positive sonograms underwent further examinations at 3, 6, 12, and 24 months of age. Complete radiological resolution was defined as at least two negative subsequent examinations, without clinical symptoms.

Clinical follow-up of the babies was performed until one of the following conditions were met:

- Reached at least 2 years of age;
- Or the complete resolution of UTD;
- Or the complete normalization of radiological findings.

All the postnatal US examinations were performed by the same pediatric radiology team. The exam was performed from 2010 to 2012 using an Esaote MyLab25Gold ultrasound machine (ESAOTE-Mailing, San Jose, CA, USA), while from 2013 to 2020 a GE Logiq S8 (GE HealthCare, Chicago, IL, USA) one was used, with a microconvex transducer and a linear transducer (7.5 MHz) in the pediatric setting.

Children with at least one abnormal US morphological criteria, such as an APRPD ≥ 14 mm, signs of severity, or bilateral UTD, underwent voiding cystourethrography (VCUG) to detect vesicoureteral reflux. Furthermore, in the case of suspected pyelo-ureteral junction (PUJ) stenosis, parenchyma reduction, and recurrent urinary tract infections, 99mTc-angioscintigraphy was performed. Children affected by complex conditions, such as PUJ stenosis, megaureters, ectopic ureter, ureterocele, posterior urethral valves, spina bifida, and prune belly syndrome, were excluded.

Informed consent was obtained verbally from the parents of the participants. This study was performed according to the Helsinki Declaration. According to the current legislation and due to the retrospective nature of this study, it did not require the local ethics committee’s approval. We retrospectively analyzed the data respecting data protection policies.

### Statistical Analysis

We report the continuous variables as median [interquartile range] and compare them in a univariate analysis by the Mann–Whitney U test. Categorical variables are reported as absolute numbers (percentage) and compared by the chi-square test (with the Fisher’s test, if appropriate).

The period from the day of birth to the last clinical evaluation available was considered the follow-up period. A univariate Cox regression analysis was performed to assess the study variables for the association with the normalization of radiological findings. Significant variables from the univariate analysis were entered into a multivariate Cox regression model to identify independent predictors of resolution. We categorized the continuous variables into dichotomous parameters (i.e., low/high) to better model hazard and fitting estimation. The optimal dividing cut-off by Youden’s index was obtained for each variable, performing a ROC curve analysis of each parameter for clinical resolution.

The survival curves were estimated by the Kaplan–Meier method. The adjusted association of factors with the clinical resolution is expressed as the hazard ratio (HR) [95% confidence interval]. A two-sided *p* ≤ 0.05 was considered significant. The statistical analysis was performed with SPSS v25^®^ (IBM, Armonk, NY, USA).

## 3. Results

From January 2010 to December 2020, 888 children with prenatal UTD and/or postnatal unilateral or bilateral UTD recorded within one month of age were evaluated in our center. Among them, 500 patients had a confirmed unilateral or bilateral UTD in two consecutive US examinations until the first month of life; 375 (75.0%) were males and 125 (25.0%) were females. The oldest patient diagnosed with UTD was 1.0 month old. The study population’s characteristics are described in Table 1.

Out of the total sample, 270 (54.0%) children had a postnatal unilateral UTD and 230 (46.0%) had a bilateral UTD. Prenatal UTD characterized 338 (67.6%) cases, while 162 (32.4%) cases were diagnosed after birth by US scans performed for other reasons. The median APRPD measured by the US at 0–2 months of age was 7.7 mm [IQR 6.0–10.0].

Regarding the delivery mode, 285 (57.0%) babies were born by vaginal delivery and 215 (43.0%) by cesarean section; 138 (27.6%) mothers were affected by comorbidities, such as endocrinopathies, hypertension, heart diseases, tumors, or neurological disorders. Among the children managed, 363 (72.6%) were term infants, while 137 (27.4%) were preterm; 431 (86.2%) had a birth weight ≥ 2500 g and 69 (13.8%) weighed < 2500 g. A small cohort of babies (29, 5.8%) was born after assisted reproduction. Of all the newborns, 9.4% presented an abnormal APGAR score (≤7) at birth. A familial history positive for kidney disease or UTD was found in six (1.2%) cases. Moreover, 13 (2.6%) newborns had associated cystic kidney disease.

During the follow-up, 86 (17.2%) children presented at least one episode of urinary tract infection (UTI). Their median APRPD at 0–2 months of age was 10.0 mm [IQR 7.0–14.3].

Ninety-nine (19.8%) children underwent VCUG, with abnormal findings in twenty-seven (27.3%) cases. The median APRPD at 0–2 months of age for children with abnormal VCUG findings was 10.0 mm [IQR 8.0–16.0]. On the other hand, 76 (15.2%) underwent 99mTc-angioscintigraphy, and 53 (69.7%) of them had an anomalous distribution of the tracer. In abnormal cases, the median APRPD at 0–2 months of age was 14.0 mm [IQR 10.0–19.5].

Finally, 11 (2.2%) patients underwent surgical treatment because of the severity of their condition. These patients’ median APRPD at 0–2 months of age was 17.0 mm [IQR 14.5–22.5].

As regards the UTD resolution, 279 (55.8%) patients had a complete resolution at the age of 6 months; an additional 55, for a total of 344 (68.8%), at 9–12 months; and 19, for a total of 353 (70.6%), at 24 months. Only 13 patients (2.6%) diagnosed with bilateral hydronephrosis had just unilateral resolution, and we managed them with the same criteria used for unresolved unilateral hydronephrosis.

**Figure 1:**
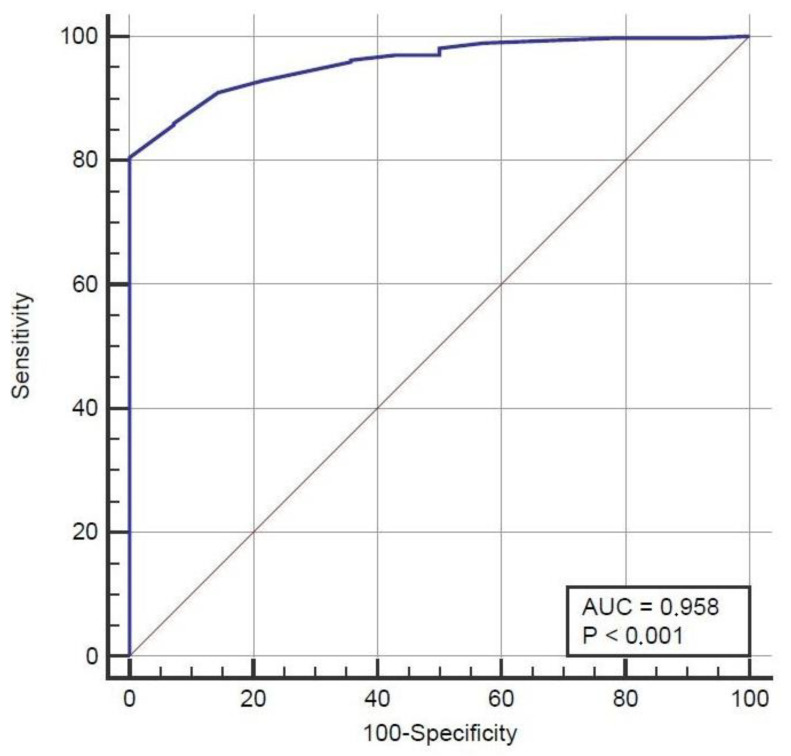
ROC curve for APRPD.

We performed a statistical analysis to investigate the differences between patients with UTD resolution and patients with persistent UTD. As for UTD resolution at 6 months of age, a statistically significant difference was found for APRPD/birth weight ratio, prenatal UTD, maximum APRPD measured at 0–2 months, bilateral UTD, and abnormal findings at VCUG and/or 99mTc-angioscintigraphy. The analysis showed a difference with statistical significance at 9–12 months of age for the APRPD/birth weight ratio, APGAR score ≤ 7, prenatal UTD, abnormal findings at VCUG and/or 99mTc-angioscintigraphy, and history of at least one episode of UTI. Finally, regarding the 24-month resolution of the UTD, significant data were found for the maximum APRPD measured. No other items with a statistically significant impact on UTD resolution were found in our analysis. An APRPD value ≤ 8.5 mm showed a sensitivity of 80.4% (95% CI [76.0–84.4]) and a specificity of 100.0% (95% CI [76.8–100.0]) in identifying candidates for spontaneous resolution within 24 months of life. The ROC curve for APRPD is presented in Figure 1.

**Figure 2:**
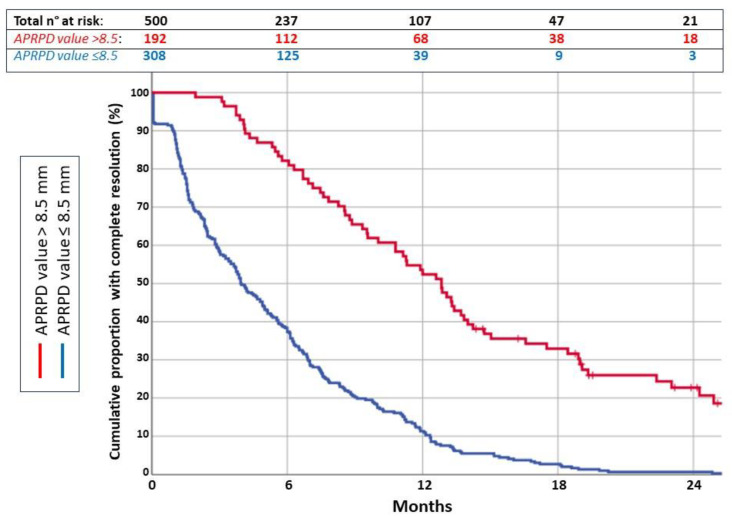
Kaplan–Meier curve for APRPD resolution during follow-up (months).

Univariate and multivariate Cox regression analyses for 24-month resolution were performed. A *p*-value < 0.05 was considered statistically significant. An APRPD value ≤ 8.5 mm was an independent prognostic factor of resolution at the age of 24 months (*p* = 0.000) (Figure 2).

Moreover, among the patients with an APRPD ≤ 8.5 mm (308 patients), we performed VCUG in 16 cases, and 8/16 were pathologic, of which 7/8 had a complete resolution at 24 months of follow-up. We also performed 99mTc-angioscintigraphy in nine cases, and 5/9 were pathologic, of which 4/5 had complete resolution at 24 months of follow-up.

## 4. Discussion

Isolated hydronephrosis is the most frequent urinary tract abnormality detected in pregnancy. Such conditions can cause significant parental anxiety related to the risk of future pathologies. A well-structured prenatal and postnatal management plan is indeed necessary [6,7,8,9,10].

Patients with CAKUTs during pregnancy often undergo extensive imaging with serial ultrasounds and sometimes magnetic resonance imaging (MRI). After birth, patients may undergo several examinations, especially periodic renal ultrasounds. If VUR is suspected, a voiding cystourethrogram (VCUG) and, in selected cases, a scintigraphy or an MRI urogram are also performed. Prenatal examinations are generally non-invasive, contrary to postnatal assessments that are more invasive and can expose the patient to radiation or require anesthesia.

Lee et al. [11], in their meta-analysis, in 2006, described that children with moderate and severe prenatal hydronephrosis had a significant risk for postnatal pathology. They suggested that comprehensive diagnostic management after birth should be performed and that mild antenatal hydronephrosis could cause a risk for postnatal UTD, requiring further prospective studies to define the best management. In the same study, the authors pointed out that patients with suspected CAKUTs should be referred to centers specialized in maternofetal medicine and that their parents should undergo prenatal counseling and should deliver in centers with units dedicated to these pathologies [11].

Coelho et al. [5], in 2007, described a population of 192 patients with severe (≥15 mm), moderate (10–14.9 mm), and mild (5–9.9 mm) isolated renal pelvic dilatation during a follow-up of 24 months. Out of 89 patients with mild dilatation, 16 (18%) presented uropathy, 27 patients (15%) required surgical intervention, and UTI occurred in 27 (14%) children. They demonstrated that the risk of uropathy and its associated morbidity was correlated with dilatation, so infants with severe dilatation should undergo comprehensive postnatal diagnostics, while, in the case of moderate dilatation, surgical correction was rarely required, and the renal pelvic dilatation frequently resolved spontaneously. Most patients with mild dilatation had no significant findings during infancy [5].

In 2011, Tombesi et al. [12] evaluated the outcome of newborns with mild isolated antenatal hydronephrosis and dilatation of 5–15 mm during the third trimester of pregnancy, confirmed by the first postnatal US exam. In their population of 193 patients, they described the total resolution of hydronephrosis at 73% during the first year, such as in our population (72.6%). They also affirmed that the VCUG and the routine antibiotic prophylaxis may not be necessary in all these children according to the observations of de Kort et al., which affirmed that, in infants with dilatation up to 15 mm, non-invasive postnatal follow-up was justified and VCUG should be performed just in cases of ureteral dilatation [13].

In the past decades, there has been a deep discussion about the relationship between VUR, UTI, and renal damage [14,15]. The importance of the early detection of VUR to reduce renal scarring has been questioned because the damage is more related to recurrent infections than to the entity of reflux [16]. The prevalence of chronic renal disease has not changed in the last decades, and according to the editorial comment of Merguerian PA, we think that renal dysplasia is the cause of most of the cases of chronic renal failure [17]. The most frequent US findings in congenital renal injury are small kidneys, cortical cysts with or without dilatation, and a thinned or hyper-echoic cortex [18,19,20]. This is a crucial point during prenatal and postnatal counseling with the parents to explain the risk of renal pathology and the type and time of follow-up.

In the past decades, there have been deep differences in the definitions and management of these conditions and clinical practice, including the methods and frequency of radiographic documentation, classification, in-utero testing, or management after birth [7,8,9,10,21,22].

We think that our study can provide clinicians with further evidence on how to approach the disease. Our findings will help in counseling families during the gestation and the follow-up of the babies. In particular, our study group aimed to find a cut-off in the measurement of the anteroposterior renal pelvis diameter that might be linked to future outcomes on renal health. We found that an APRPD value ≤ 8.5 mm showed a sensitivity of 80.4% and a specificity of 100.0% in identifying candidates for spontaneous resolution within 24 months of life. According to our analyses, 8.5 mm can be used as a cut-off to reassure parents and clinicians of the benignity of the postnatal dilatation; in the case of higher pelvis dimensions, the child should undergo a structured follow-up.

Furthermore, during the follow-up, risk factors for the persistence of dilatation were identified in the presence of prenatal UTD, such as low birth weight and mal adaptation at birth (APGAR score ≤ 7), meaning that, the healthier the baby and the more regular the perinatal course, the more the APRPD tended to resolve spontaneously. Low birth weight in particular has been associated with the delayed maturation of renal structures and impaired nephron development, which may contribute to the persistence of UTD [23]. Specifically, infants with low birth weight are more likely to exhibit reduced renal functional reserve and diminished compensatory mechanisms, potentially exacerbating urinary stasis or obstruction [23]. Additionally, a low APGAR score, indicative of perinatal hypoxia, may compound these effects by increasing the risk of ischemic injury to the developing kidney. Longitudinal monitoring of renal function and imaging in infants with low birth weight or abnormal APGAR scores is, therefore, essential to predict and manage the resolution or progression of UTD effectively.

During the follow-up, 17.2% of the children presented at least one episode of urinary tract infection (UTI). UTIs were directly linked to the persistence of renal pelvis dilatation at 9–12 months, as already discussed by Oliveira et al. [2] and Mahant et al. [24]. Antenatal and postnatal APRPD and UTD are, in fact, known risk factors for UTI.

Abnormal findings at VCUG and/or 99mTc-angioscintigraphy were significantly related to renal pelvic dilatation at the US scans, meaning that the presence of a congenital anomaly is not just a morphological characteristic but also affects the function of the kidney itself proportionately. If abnormalities are identified through VCUG or 99mTc-angioscintigraphy, the recommended treatments and management strategies are tailored to the underlying pathology and its severity [4,12,13,25]. In the case of VUR, management depends on the reflux grade. Low-grade VUR is typically managed conservatively, while higher grades (III-V) may necessitate surgical intervention, such as ureteral reimplantation or endoscopic injection of bulking agents [4,12,13]. In the case of severe obstruction detected by angioscintigraphy, surgical intervention is once again indicated [25]. If there is reduced renal perfusion or function, it is important to preserve residual renal function. Therefore, these exams provide essential insights into the severity and functional impact of urinary abnormalities, ensuring that treatment plans are personalized to prevent complications and preserve renal function.

The accuracy of this study relies on many points. First, the timing of the US scans, especially the first one performed. As we explained in the methods section, the study population underwent a urinary tract ultrasound examination within the first month after birth. It is important to delay the US at least until after the first 48 h of life because of the physiologic weight loss and oliguria in the newborn as discussed by Liu et al. [25], in order to not underestimate the possible APRPD. Second, the scans were performed by the same radiologist every time, avoiding the interobserver variability that usually affects US scans. Third, our sample was quite large, being from the Spina Bifida and Congenital Uropathies Outpatients Center based in a hospital with more than 4000 births every year, and the observation was also carried on for a long time in case of non-resolution.

The limitations of our study include the variability in the length of follow-up, depending on drop-offs chosen by some parents, the level of hydration of the patient and/or bladder (filled or empty) during the US examination, the variability existing in the literature on the APRPD definitions, and the inter- and intraobserver variation for all grading systems. As regards the last point, we used the definition given by Coelho et al. [5].

## 5. Conclusions

Our study provides further evidence on how to approach these conditions, in particular, to identify candidates for spontaneous resolution and reassure families. The social health care costs and the efficacy of routine radiological examinations as a screening tool for the potential progression of pathologies, like hydronephrosis, remain controversial. The data from our study and similar ones conducted on the same pathology are fundamental because they allow us to identify patients at risk of disease progression who should, therefore, be recipients of more in-depth examinations and treatments.

## Figures and Tables

**Table 1:** Characteristics of the study population.

	Total (500)	M (375)	F (125)	*p*-Value
Age at diagnosis (months)	0.10 [0.03–0.46]	0.10 [0.03–0.47]	0.10 [0.03–0.53]	0.752
Gestational age	39.0 [37.0–40.0]	39.0 [37.0–40.0]	39.0 [37.0–39.5]	0.857
Birth weight (kg)	3.20 [2.80–3.54]	3.21 [2.81–3.55]	3.20 [2.72–3.52]	0.653
Follow-up time (months)	5.46 [2.07–11.18]	5.80 [2.27–11.20]	3.93 [1.60–11.18]	0.092
APGAR 1’	9.00 [9.00–9.00]	9.00 [9.00–9.00]	9.00 [8.00–9.00]	0.062
APGAR 2’	10.00 [9.00–10.00]	10.00 [9.00–10.00]	10.00 [9.00–10.00]	0.399
APRPD * at 0–1 month (mm)	7.65 [6.0–10.00]	8.00 [6.0–10.00]	7.00 [5.60–12.00]	0.821
Bilateral renal pelvis dilatation	230 (46.0%)	175 (46.7%)	55 (44.0%)	0.604
Preterm	137 (27.4%)	100 (26.7%)	37 (29.6%)	0.524
Low birth weight (<2.50 kg)	69 (13.8%)	49 (13.1%)	20 (16.0%)	0.410
APGAR ≤ 7	45 (9.4%)	33 (9.1%)	12 (10.2%)	0.727
Cesarean delivery	215 (43%)	161 (42.9%)	54 (43.2%)	0.958
Assisted reproduction pregnancy	29 (5.8%)	23 (6.1%)	6 (4.8%)	0.851
Maternal comorbidities	138 (27.6%)	113 (30.1%)	25 (20.0%)	0.028
Family history of kidney disease	6 (1.2%)	3 (0.8%)	3 (2.4%)	0.155
Antenatal renal pelvis dilatation	338 (67.6%)	256 (68.0%)	83 (66.4%)	0.768

* APRPD: anteroposterior renal pelvis diameter.

## Data Availability

Data available on request due to privacy restriction.
